# Cultural Influences on Typical Gross Motor Development in Early Childhood: A Scoping Review

**DOI:** 10.1111/cch.70364

**Published:** 2026-10-15

**Authors:** Jessica A. Silveira, Megan B. Flores, Jessica Barreca, Rebecca C. Hyde

**Affiliations:** ^1^ Department of Physical Therapy Texas State University Round Rock Texas USA; ^2^ Department of Physical Therapy Baylor University Waco Texas USA; ^3^ Program in Physical Therapy Saint Louis University St. Louis Missouri USA; ^4^ Saint Louis University St. Louis Missouri USA

**Keywords:** caregiver practices, culture, motor development, scoping review

## Abstract

**Background:**

Guided by the developmental niche and integrated ecological frameworks, cultural practices (e.g., caregiving routines, parental beliefs and contextual affordances) may be associated with the timing and expression of motor milestones. However, the extent and nature of these associations worldwide remain unclear. How this literature defines typical development and conceptualizes culture is similarly unclear. The aim of this scoping review was to explore associations between cultural practices and typical gross motor (GM) development among children aged 0–5 years around the world, and map how included studies defined typical development, operationalized culture and studied these relationships.

**Method:**

Using the approach outlined by the Joanna Briggs Institute and in alignment with the Preferred Reporting Items for Systematic Reviews and Meta‐Analyses extension for Scoping Reviews (PRISMA‐ScR) an a priori protocol was developed for this scoping review. A comprehensive search of APA PsycINFO (OVID & EBSCO), CINAHL Ultimate, ERIC, ProQuest Dissertations & Theses Global, PubMed, Scopus and Web of Science was conducted for English‐language publications from 1994 to April 2025. Eligible studies examined cultural practices related to typical GM development in children aged 0–5 years. Data were extracted on cultural variables, definitions of typical development, GM outcomes and reported facilitators and barriers.

**Results:**

A total of 166 publications were included from 8145 screened records. Frequently examined cultural variables included geographic location (*n* = 109), socioeconomic status (SES) and home affordances (*n* = 105), ethnicity/race (*n* = 103) and social setting (*n* = 87). Higher SES, enriched home affordances, parental knowledge and expectations and access to preschool motor curricula were associated with more advanced GM development, consistent with an integrated ecological perspective. Limited space or resources, lower socioeconomic conditions, restrictive caregiver beliefs and reduced outdoor activity were associated with lower GM performance. Definitions of ‘typical development’ were inconsistent, with only 24 studies providing explicit criteria, and culture was operationalized variably across studies. The evidence base was predominantly cross‐sectional.

**Conclusion:**

Cultural context is consistently associated with variation in early GM development. Findings support interpreting motor performance through an integrated ecological lens, using culturally validated assessment tools to reduce misclassification of typical variation as delay and prioritizing longitudinal, globally representative research to inform equitable developmental surveillance and intervention planning.

## Summary

- Gross motor development should be interpreted within the child's cultural, caregiving and environmental context rather than using universal assumptions of typical development.
- Clinicians should consider caregiver beliefs, opportunities for movement, home affordances and access to early childhood settings when assessing young children's motor development.
- Use of motor assessments normed primarily in Western populations may risk misclassifying culturally typical variation as developmental delay; culturally validated tools and context‐sensitive interpretation are needed.
- Future research should prioritize longitudinal, globally representative and mixed‐methods studies to clarify how cultural practices, social conditions and policy environments shape early gross motor trajectories.

## 1 Introduction

Child development is a multifaceted process influenced by a dynamic interplay of genetic, environmental and cultural factors. Gross motor (GM) skill acquisition, including motor milestone trajectory and timing, can be affected by factors such as geographic region (Nobre et al. 2020; Bardid et al. 2015), socioeconomic status (SES) (Brian et al. 2019; Valentini et al. 2015; Logan et al. 2015), population density (Niemistö et al. 2019) and caregiver support (Barnett et al. 2019; Valentini et al. 2020). GM development encompasses movements using large muscle groups (e.g., rolling, sitting, crawling, walking, running and jumping) (Adolph and Hoch 2019; Berger et al. 2021). Research consistently reports associations between cultural contexts and the progression and acquisition of GM milestones (Berger et al. 2021; Karasik et al. 2010; Cintas 1989; Karasik et al. 2015). Understanding how culture influences GM development is crucial for clinicians, educators and policymakers aiming to foster optimal developmental outcomes across diverse populations (Pasick et al. 1996).

The US Centers for Disease Control (CDC) defines culture as ‘group membership, such as racial, ethnic, linguistic or geographical groups, or as a collection of beliefs, values, customs, ways of thinking, communicating and behaving specific to a group’ (US Centers for Disease Control and Prevention 2024). In this review, culture was operationalized broadly to include both culturally embedded beliefs and practices (e.g., caregiving routines, parental beliefs and developmental expectations) and group‐ or context‐level variables commonly used in the literature as proxies for cultural context (e.g., geographic location, ethnicity/race, SES and home affordances). Cultural practices, encompassing caregiving routines, parental beliefs and everyday experiences, are closely linked to children's motor environments and developmental trajectories (Berger et al. 2021; Super and Harkness 1986). The influence of extrinsic factors on child development is grounded in several contemporary theories of development, including the dynamic systems theory (Thelen and Smith 1994), ecological theory (Bronfenbrenner 1979) and developmental niche framework (Harkness and Super 1994). Dynamic systems theory broadly frames development as a result of reciprocal interactions between multiple intrinsic and extrinsic factors (Thelen and Smith 1994), while ecological theory explicitly emphasizes the role of environmental context (e.g., home affordances, sociocultural context, caregiving practices) in development (Bronfenbrenner 1979). The developmental niche framework emphasizes considering the impact of parental psychology, culturally informed caregiving practices and home affordances (e.g., the availability of physical space, toys and social opportunities in the home that support motor development) when assessing a child (Harkness and Super 1994). Our study is grounded in the integrated ecological model of motor development proposed by An and Libertus (An and Libertus 2025). This novel theoretical framework blends the ecological theory and the developmental niche framework while explicitly addressing motor development and emphasizing parental perspectives. Central to this model is the focus on the parent–child dyad within their unique sociocultural context (An and Libertus 2025).

A study comparing early infancy in the United Kingdom and Uganda found cultural differences in parental behaviours, such as maternal proximity and the social distribution of caregiving, with corresponding differences in children's motor trajectories (Holden et al. 2022). Differences in parental beliefs about motor development have also been identified between different Western cultures, potentially impacting parental caregiving practices such as motor activity stimulation (van Schaik et al. 2018; Perkins et al. 2025). Parents' culturally informed beliefs regarding child development can play a crucial role in influencing caregiving practices and shaping motor development. Parental perceptions of motor development timelines and values placed on motor competencies vary across cultures and can influence the frequency and type of motor stimulation provided to young children (Adolph and Hoch 2019; Karasik et al. 2015). A study examining motor skills of 12‐month‐olds across three different cultures found motor development was influenced by both direct and indirect caregiving practices (Wang et al. 2025). Another study comparing sitting proficiency among 5‐month‐olds across six countries found substantial cultural differences in parental behaviours that were associated with the proficiency of infant motor skills (Karasik et al. 2015).

The evolving research landscape includes more culturally diverse samples, enhancing our understanding of how culture influences motor development. However, a gap remains in understanding the extent to which culture is associated with typical GM development worldwide. In addition to identifying reported cultural influences on GM development, it is also important to examine how this literature has defined typical development, conceptualized culture and studied these relationships methodologically. Munn et al. describe utilizing a scoping review to identify gaps in knowledge, explore a body of literature, or clarify concepts (Munn et al. 2018). In scoping reviews, the research questions are broader, emphasizing the comprehensiveness and breadth of literature without necessarily assessing evidence quality (Anderson et al. 2020). Alternatively, systematic reviews identify and retrieve evidence toward a specific question and, through appraisal and synthesis of results, make recommendations (Munn et al. 2018). Therefore, a scoping review was identified as an appropriate methodology to map the existing literature and identify research gaps for clinicians and researchers (Arksey and O'malley 2005). Mapping research methods and measurement approaches is particularly relevant in a scoping review because methodological variability may shape how cultural influences are identified, interpreted and compared across studies. Accordingly, the research questions were developed to address identified conceptual, methodological and contextual gaps in the literature and to guide a comprehensive examination of the available evidence.

This scoping review aims to address the overarching question: How do current cultural practices impact typical GM development worldwide among children aged 0–5 years? Specifically, the following questions were developed to address conceptual, methodological and contextual gaps in the literature:

1. How do different cultures define ‘typical’ motor development?
2. What research methods are currently used to investigate the connection between cultural context and GM development?
3. Which culturally embedded practices and broader contextual variables have been examined in relation to GM development, and how are they characterized across studies?
4. What facilitators and barriers to GM development exist across different cultural contexts?

By exploring this topic within the key conceptual elements of population (children 0–5 years), concept (GM development) and context (culture), this review seeks to inform culturally sensitive clinical practices and guide future research.

## 2 Methods

### 2.1 Protocol Registration

An a priori protocol was developed, aligned with the Joanna Briggs Institute (JBI) and the Preferred Reporting Items for Systematic Reviews and Meta‐Analyses extension for Scoping Reviews (PRISMA‐ScR) checklist (Tricco et al. 2018; Peters et al. 2020) (Appendix S1). The project was registered in Open Science Framework (OSF) prior to starting (DOI: https://doi.org/10.17605/OSF.IO/AHQDB). No funding was obtained for this research and the authors have no known conflicts of interest.

### 2.2 Sources of Evidence and Search Strategy

A research librarian (RH) assisted in the search by identifying pertinent publications in these databases: EBSCO's CINAHL Ultimate (EBSCO), APA PsycINFO (OVID & EBSCO), PubMed, ERIC (EBSCO), Scopus, Web of Science Core Collection, and ProQuest Dissertations & Theses Global. The search was run in November 2023, then re‐run in March 2025 to ensure the findings were current.

The search strategy followed the PCC method (Table 1) using a combination of keywords, subject headings and medical subject headings (MeSH). Publication years were limited to 1994 or after in alignment with the ‘Back to Sleep’ campaign (now ‘Safe to Sleep’) initiated in the United States (US) by the Eunice Kennedy Shriver National Institute of Child Health and Human Development (NICHD)in 1994 (Development EKSNIoCHaH, n.d.). This campaign recommended babies be placed on their backs to sleep to reduce the risk of Sudden Infant Death Syndrome (SIDS) and was based on studies published in the Netherlands (1988), Australia (1991), New Zealand (1991) and the United Kingdom (1991) that found links between SIDS and stomach sleeping (Development EKSNIoCHaH, n.d.). As this global policy represents a significant shift in caregiving practices and its potential influence on developmental outcomes, the authors chose to include articles published which followed the launch of the 1994 campaign. To capture early GM development, articles with participants 0–5 years were included. Meta‐analyses, systematic reviews and other secondary sources were included initially; however, they were excluded from data extraction. The librarian (RH) hand‐searched the reference lists of these publications to identify relevant articles initially missed. See Appendix S2 for the full electronic search strategies.

**TABLE 1:** PCC method and relevant terms.

Population	Children; paediatric; infants; toddlers; young children; 0–5 years early childhood
Concept	Motor development; gross motor development; walking; crawling; creeping; sitting; rolling; ambulation; typical development; motor milestones; motor skills; motor development variability; mobility
Context	Culture; cultural practices; cultural considerations; cultural beliefs; cultural values; customs; traditions; ethnicity; race; parental or caregiver expectation; parental or caregiver practices; parental or caregiver handling

### 2.3 Process of Study Selection

The scoping review was performed using Covidence (Veritas Health Innovation, Melbourne, Australia), a software management program for systematic reviews. The settings allowed for reviewer blinding during study selection (Peters et al. 2020; Peters et al. 2024). An iterative piloting process was performed to determine search criteria and reviewer reliability. During the first pilot, the search terms and criteria were clearly defined through CINAHL. Two reviewers (JS and JB) independently screened the titles and abstracts of 100 articles for possible inclusion. A third reviewer (MBF) reviewed conflicts. A 75% minimum agreement between the two independent reviewers was set and exceeded before proceeding.

The study selection and data extraction process was refined through a second pilot with an updated CINAHL search strategy. The same process was performed with the same two reviewers (JS and JB) screening 100 article abstracts for possible inclusion and noting any reasons for exclusion. The 75% agreement threshold was exceeded, and full text publications of selected abstracts were imported into Covidence. The three‐reviewer method was used for screening 10 full text articles. Preselected reasons for exclusion were created and imported into Covidence based on notes for exclusion from the first pilot.

The search aimed to identify all relevant publications, which described typical cultural practices related to GM development without interventions. Studies were included only if child outcomes were reported. UNESCO defines culture as ‘the set of distinctive spiritual, material, intellectual and emotional features of society or a social group … [which] encompasses, in addition to art and literature, lifestyles, ways of living together, value systems, traditions and beliefs’ (Napier et al. 2017). Aspects of culture included, but were not limited to, ethnicity/race, religion, geography, linguistics, social and socioeconomics. For this scoping review, culture was intentionally operationalized broadly to capture both culturally embedded beliefs and caregiving practices as well as contextual factors that shape children's developmental environments. Accordingly, variables such as SES and home affordances were considered contextual factors that may intersect with culture, rather than inherently culture‐specific constructs.

Articles were excluded for these reasons: published before 1994, outside age range, no cultural reference, not typical development, not about GM development, focus is on physical activity, intervention or intervention protocol, wrong population (not about the child), review/meta‐analysis/book, unable to find full text, no English translation available and ‘other’. For this review, GM development referred to the acquisition, timing and performance of age‐related motor skills and milestones involving large muscle groups, whereas physical activity referred to general movement participation or activity levels. Studies focused primarily on physical activity behaviour rather than GM skill development or milestone attainment were therefore excluded. Data extraction was completed on three full text articles by the two reviewers with discussion among reviewers to resolve inconsistencies.

After the two pilots demonstrated consistent agreement above 75%, a new folder was created for the final review. The librarian searched preselected databases and uploaded titles and abstracts of all relevant publications using the search terms. The three‐reviewer method was used for the final review.

### 2.4 Data Extraction and Analysis

Two reviewers (JS and JB) performed pilot testing of the data extraction template. Modifications were made to improve consistency in data extraction, and the final version was pilot tested by the same two reviewers prior to implementation. Each article was extracted by one reviewer due to the high volume of included publications. Bibliometric variables extracted included: author(s), publication year, study design, purpose/aim of study, number of participants and participant ages. Cultural variables extracted included: culture definition, definition of culture, cultural group, cultural aspect, definition of ‘typical’ GM development, GM aspect, caregiver practice, GM assessment measure, results of study, motor development facilitators and barriers to motor development. For the purposes of data extraction, ‘facilitators’ and ‘barriers’ denote factors reported by primary authors as positively or negatively associated with GM outcomes; these labels do not imply causal relationships. Frequencies of concepts, characteristics and relevant details were reported. The data were charted and reviewed by all authors.

## 3 Results

In November 2023, the initial search of databases yielded 11 306 publications. After removing 3686 duplicates, 7620 remained for screening. To ensure current findings, a March 2025 follow‐up search yielded 1109 additional records. After removing 426 duplicates, 683 publications remained for screening. During title and abstract screening, 158 duplicates were removed. A total of 8145 records were screened with 7676 excluded. Of the 469 articles retrieved for full‐text review, 302 were excluded resulting in 166 records for the final review. The PRISMA Flow diagram of the search, screening and selection process is shown in Figure 1. All publications with extracted data are shown in Appendix S3.

**FIGURE 1:**
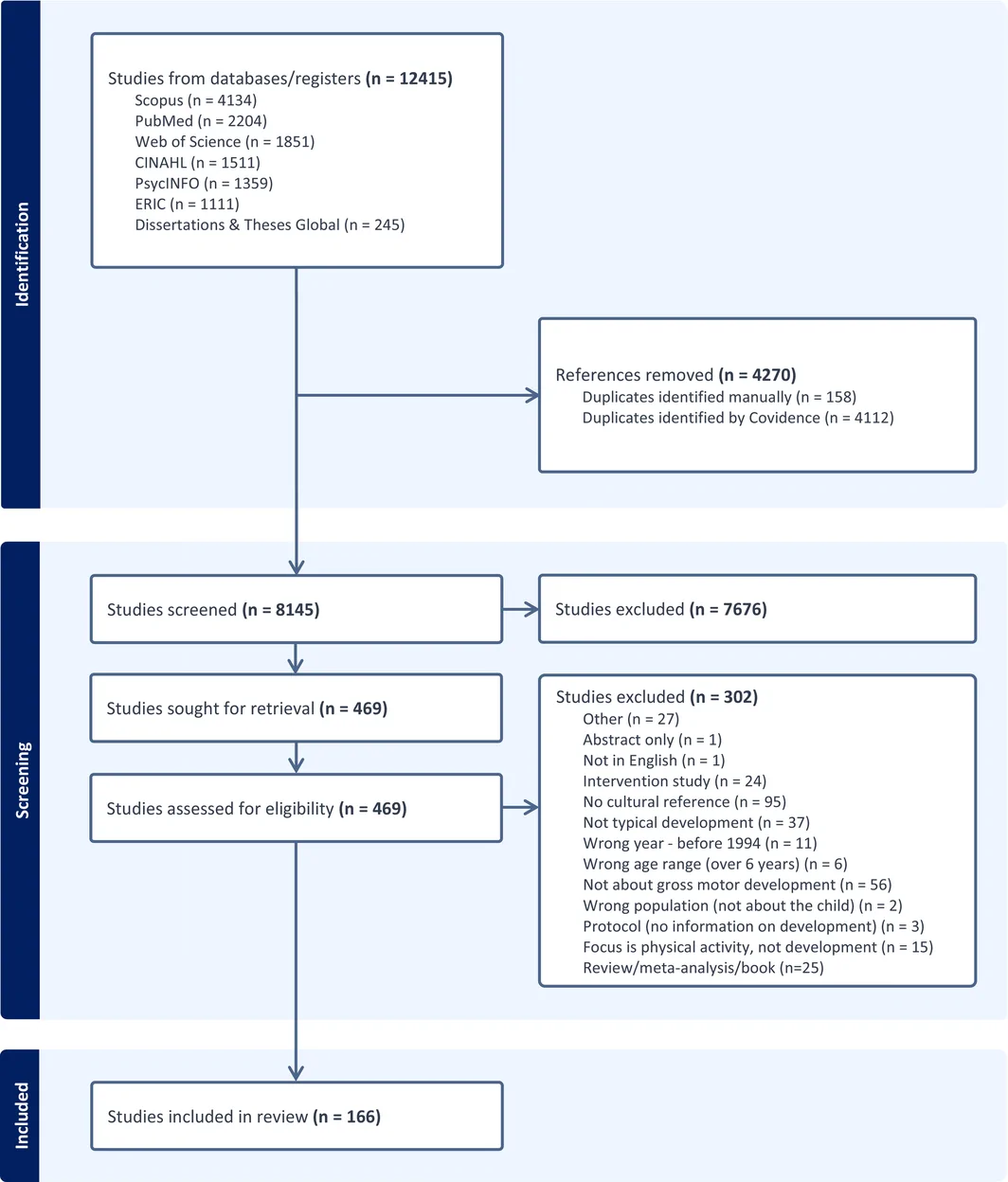
PRISMA flow diagram of the identification, screening, eligibility assessment and inclusion of studies in the scoping review.

Most included articles (*n* = 68) were published between 2010 and 2019. Notably, 60 articles were published in the last 5‐year period, demonstrating increased research activity. Between 2000 and 2009, only 33 articles were published, and five between 1995 and 1999.

### 3.1 Study Designs

The connection between culture and GM development was primarily investigated through cross‐sectional studies (*n* = 133). A small portion of articles used a longitudinal study design (*n* = 30). One article reported findings for multiple studies and included both cross‐sectional and longitudinal designs. Quantitative analysis was primarily used to investigate the impact of culture on GM development (*n* = 164), with one study implementing a mixed methods design and one qualitative study.

### 3.2 Cultural and Contextual Variables Investigated

An array of variables was investigated, with many publications examining the impact of multiple cultural and contextual variables on GM development. The most frequent cultural and contextual variables investigated were geographic location (*n* = 109), SES/home affordances (*n* = 105), ethnicity or race (*n* = 103) and social setting (e.g., attending daycare) (*n* = 87). Other variables investigated included language (*n* = 8) (Saraiva et al. 2013; Saccani et al. 2016; Ertem et al. 2018; Kelly et al. 2006; Eyre et al. 2018; Cools et al. 2011; Jelsma and Ferguson 2007; Kerfeld et al. 1997), age and education level of parent(s) (*n* = 4) (Tella et al. 2018; Kolling et al. 2014; Giagazoglou et al. 2007; Kappelt et al. 2024), child gender (*n* = 3) (Starrett et al. 2022; Richards et al. 2022; Kamat 2013), religion (*n* = 1) (Cools et al. 2011), United Nations (UN) Human Development Index (HDI) (*n* = 1) (Gontijo et al. 2014) and food security (*n* = 1) (Milner et al. 2018).

### 3.3 Defining Typical Development

Motor development is variable between individual children within the same culture and among children in different cultural settings. Most included articles did not define ‘typical’ GM development. Twenty‐four articles provided an explicit definition of typical development, 23 of which used cutoff scores, normative scores or a percentage of children able to complete the motor skill. Only one article (Yalçin et al. 2012) provided a definition using the motor skill of walking by stating: ‘Healthy infants begin to walk around 12 months with some biological variability. By the age of 18 months, all healthy term infants attain walking ability [pg. 260]’.

### 3.4 Measures of Gross Motor Development

Gross motor development of participants was measured using a broad range of tools. This includes both direct formal assessment by trained professionals and informal assessment from parents or other caregivers, incorporating both structured and unstructured assessment methods. Standardized assessments (*n* = 108) and developmental screens (*n* = 31) were the most used tools in the included studies. Other tools included standardized questionnaires (*n* = 4) (Wu et al. 2012; Celhay et al. 2020; Playford et al. 2017; Smith 2021), nonstandardized questionnaires (*n* = 7) (Yalçin et al. 2012; Chiang et al. 2015; Stanitski et al. 2000; He et al. 2015; Nelson et al. 2004; Shiva et al. 2010; Policastro et al. 2024), developmental checklists (*n* = 4) (Angulo‐Barroso et al. 2011; Prado et al. 2017; Larson et al. 2017; WHO Multicentre Growth Reference Study Group et al. 2006), observation (*n* = 4) (Karasik et al. 2015; Wang et al. 2025; Karasik et al. 2022; Aime et al. 2021) and a nonstandardized gross motor assessment (*n* = 1) (Morales et al. 2023). Eight studies used a multimodal approach to measure gross motor development, including the following combinations: nonstandardized questionnaire and observation (*n* = 1) (Wyckoff 2010); developmental screen, nonstandardized questionnaire and semi‐structured interview (*n* = 1) (Barlow and Reynolds 2018); standardized assessment and developmental checklist (*n* = 1) (Karasik et al. 2023); standardized assessment and observation (*n* = 2) (Hixon 2002; Graciosa et al. 2024); standardized assessment and standardized questionnaire (*n* = 1) (Brian et al. 2018); and standardized assessment and developmental screen (*n* = 2) (Lejarraga et al. 2002; Novakoski et al. 2023).

### 3.5 Cultural and Contextual Facilitators and Barriers for Motor Development

#### 3.5.1 Geographical

Living in a developed country or in a country with higher health and education outcomes was associated with higher GM performance. One article (Salah El‐Din et al. 2021) reported higher GM development in children living in developed countries compared to those in developing countries, while another found that a lower HDI was associated with lower GM development (Gontijo et al. 2014). The UN HDI is a composite measure of human development, considering health and life expectancy, education and national income (Programme UND, n.d.).

Mixed results were reported regarding associations between geographical density and GM development. Two articles found an urban living environment to be a facilitator (Starrett et al. 2022; Richards et al. 2022), while another article found increased GM development in a rural environment (Santamaría‐Vázquez et al. 2016). A fourth article reported higher population density to be a barrier (Niemistö et al. 2019).

Four articles reported higher GM development in young children living in a country that integrated physical education (Brian et al. 2018; Van Waelvelde et al. 2008; D'Hondt et al. 2019) or emphasized motor development in preschools (Richards et al. 2022; Giagazoglou et al. 2005). Three articles compared motor development between Belgium and either the US or Greece, finding greater GM ability in Belgian children where preschools included physical education (Brian et al. 2018; Van Waelvelde et al. 2008; D'Hondt et al. 2019). Giagazoglou et al. found Greek children scored higher than the British reference sample in GM, noting the emphasis Greek preschools placed on motor development (Giagazoglou et al. 2005).

#### 3.5.2 Socioeconomic Status and Home Affordances

Higher SES and greater affordances within the home were associated with higher GM ability in many studies. Twelve articles reported that higher SES was associated with higher GM skills (Starrett et al. 2022; Celhay et al. 2020; Black et al. 2016; de Paiva et al. 2010; Valadi and Gabbard 2020; Valadi 2022; Cesário Defilipo et al. 2021; Dai et al. 2022; Lima et al. 2004; Correia et al. 2019; Pereira et al. 2016; Saccani and Valentini 2013), while one study (Gao et al. 2024) found that children from lower SES homes had higher GM ability. Greater home affordances were also positively associated with GM ability (*n* = 9) (Black et al. 2016; Valadi and Gabbard 2020; Lima et al. 2004; Lovison et al. 2021; Santos et al. 2001; Cao et al. 2022; Kolobe et al. 2006; Hossain et al. 2024; Kaur and Randhawa 2021). Facilitators included adequate space for GM activities (Kaur and Randhawa 2021; Chen et al. 2003), a greater amount and variety of toys and play materials (Prado et al. 2017; Cesário Defilipo et al. 2021; Kaur and Randhawa 2021; Koshy et al. 2021; Deshpande and Metgud 2023; Kavousipor et al. 2021; Bornstein et al. 2005), more people in the home available to play with the child (de Paiva et al. 2010), public transportation use (Chow et al. 2001) and outdoor movement affordances (Wyckoff 2010; Giagazoglou et al. 2005; Jin et al. 2016). Home affordances were often linked to SES due to increased home space and play material availability.

#### 3.5.3 Caregiver Characteristics

Parental education levels, beliefs, expectations and knowledge about motor development were associated with GM development. Higher parental education levels were associated with higher GM ability in 14 articles (Cools et al. 2011; Kappelt et al. 2024; Yalçin et al. 2012; Novakoski et al. 2023; Black et al. 2016; Valadi 2022; Saccani and Valentini 2013; Santos et al. 2001; Kaur and Randhawa 2021; Wrigglesworth et al. 2023; Syrengelas et al. 2014; Fink et al. 2020; Kappelt et al. 2024; Saccani and Valentini 2012). Other facilitators included maternal knowledge about early development (*n* = 1) (Cuartas et al. 2020), parental perception of child competence (*n* = 1) (Chiang et al. 2015), high value placed on sports‐specific physical activity (*n* = 1) (Cools et al. 2011) and higher parental expectations (*n* = 3) (Graciosa et al. 2024; Kolobe et al. 2006; Lee 2022). Parental belief that early GM development does not require active stimulation (*n* = 1) (Graciosa et al. 2024), parent's lack of knowledge of early motor development (*n* = 2) (Salah El‐Din et al. 2021; Lee 2022) and negative parent attitude about outdoor activities (*n* = 1) (Jin et al. 2016) were suggested as barriers to GM development. Finally, being a single mother was associated with slower GM development (*n* = 1) (Paterson et al. 2011) while maternal social support (*n* = 1) (Salah El‐Din et al. 2021) and paternal involvement (*n* = 1) (Kato et al. 2024) were positively associated with motor development.

#### 3.5.4 Caregiving Practices

Caregiving practices that provided opportunities for a young child to move and engage with a caregiver were positively associated with GM skills. In infants, this included reduced frequency in supine (*n* = 1) (Graciosa et al. 2024), a higher frequency of floor play (*n* = 1) (Graciosa et al. 2024), increased time creeping (*n* = 1) (Wyckoff 2010), reduced time being carried (*n* = 1) (Wyckoff 2010), playing in sitting with or without support (*n* = 1) (Deshpande and Metgud 2023) and placement in different positions (*n* = 1) (Pereira et al. 2016). Greater duration of exclusive breastfeeding was also associated with better GM development (*n* = 2) (Angulo‐Barroso et al. 2011; Sabri et al. 2022). Across the first 6 years of childhood, more time spent engaging and playing with caregivers (*n* = 8) (Chiang et al. 2015; Prado et al. 2017; Correia et al. 2019; Saccani and Valentini 2013; Deshpande and Metgud 2023; Kavousipor et al. 2021; Fink et al. 2020; Cuartas et al. 2020) and increased freedom to explore the environment (*n* = 5) (Kerfeld et al. 1997; Deshpande and Metgud 2023; Bornstein et al. 2005; Charafeddine et al. 2013; Tupsila et al. 2020) were associated with more advanced GM development. Other caregiving practices found to be positively associated with motor development included regular family hikes (*n* = 1) (Berg et al. 2008), early introduction to ball games (*n* = 1) (Chow et al. 2001), older sibling as a caregiver (*n* = 1) (Kerfeld et al. 1997), daily exposure to dance (*n* = 1) (Goodway et al. 2010), biking to school (*n* = 1) (Cools et al. 2011) and extended family living in the home (*n* = 1) (Tupsila et al. 2020).

## 4 Discussion

Historically, research regarding motor development has been based on samples from Western, Educated, Industrialized, Rich and Democratic (WEIRD) cultures (Henrich et al. 2010). As scholarship has broadened to include more culturally diverse populations, the field has gained a more refined understanding of how cultural contexts shape early motor development. This scoping review contributes to that body of work by synthesizing current evidence on how culture influences typical GM development across global settings. Our study aimed to explore how different cultures define ‘typical’ GM development, identify predominate research methods used to investigate the connections between cultural context and GM development, determine which culturally embedded practices and contextual variables have been examined in relation to GM development and identify facilitators and barriers to GM development across cultural contexts. Our findings reflect a marked rise in publications on this topic since 2010, emphasizing a growing recognition of culture as a meaningful developmental factor. Adolph and Hoch (Adolph and Hoch 2019) assert that ‘motor development is enculturated’, noting that social and cultural forces shape children's motor behaviours. Harkness and Super's developmental niche framework (Harkness and Super 1994) highlights culturally regulated customs of caregiving as central to development. And An and Libertus (An and Libertus 2025) integrated ecological model emphasizes the influence of parental perspectives and the transactional relationship between parents and children within their sociocultural context on motor development. Consistent with these perspectives, our review found substantial emphasis on cultural and contextual influences in studies of typically developing children. However, we also observed considerable variability in how ‘typical’ development was defined and noted few studies explicitly defined ‘culture’, indicating conceptual inconsistency across the literature.

A notable finding of this review was the inconsistent operationalization of ‘typical’ GM development across studies. Although motor development is inherently variable both within and between cultural contexts, only a small subset of included studies provided explicit criteria or definitions of typical development, often relying on cut‐off scores, normative references or percentages of children achieving specific milestones. Most studies did not define typical development, thereby implicitly assuming a universal developmental trajectory. Additionally, many investigations relied on standardized motor assessments normed primarily on Western populations, often without evidence of cultural adaptation or validity in the local population. This reflects a broader concern regarding the dominance of WEIRD samples in studies of child development (Henrich et al. 2010). Evidence indicates that developmental norms are embedded within cultural contexts. Therefore, applying standards developed from WEIRD samples across culturally diverse contexts may result in misclassification of cultural variation as developmental delay while overlooking meaningful differences in developmental trajectories. The lack of culturally grounded definitions of typical GM development represents a critical gap in the literature, emphasizing the need for culturally responsive assessments and normative frameworks that reflect diverse cultural settings.

Cross‐sectional study design and quantitative analysis were the primary research methods used in the included studies. As a result, most included studies examined associations or differences between GM development and cultural and contextual variables at a specific moment in time. The limited use of longitudinal studies limits the depth of interpretation from the studies, as predictive relationships cannot be established and comparisons between developmental trajectories are not possible in cross‐sectional studies. Additionally, the lack of qualitative data limits the conclusions that can be drawn, especially about caregiver's perspectives and beliefs surrounding caregiving within their cultural context. GM development was primarily measured using standardized outcome measures and developmental screens; however, measurement tools were not consistently validated for the cultural context and included parent‐reported tools, standardized assessments administered by trained professionals, or a combination of both. Although previous research has highlighted strengths associated with parent‐reported assessments of young children's gross motor skills (Federico et al. 2021), parent reports may be influenced by limited training in motor development and potential bias related to evaluating their own child, making these measures more subjective in nature. In contrast, gross motor assessments administered by trained clinicians or early childhood professionals are considered more objective (Zysset et al. 2018). The variations in assessment methods may influence how study outcomes are interpreted and compared across this scoping review.

The findings of this review identified a heavy reliance on Western‐normed motor assessments, such as the Test of Gross Motor Development–2 (TGMD‐2), Peabody Developmental Motor Scales–2 (PDMS‐2), Bruininks–Oseretsky Test of Motor Proficiency–2 (BOT‐2) and the Griffiths Scales. These measures were frequently applied across diverse cultural contexts, often without clear evidence of local norming or psychometric validation. Although some studies (Valentini 2012; Song et al. 2022) conducted formal cross‐cultural adaptation or validation, these efforts are limited in comparison to the extensive global application of these assessments. When considered alongside the inconsistent definitions of ‘typical’ development and frequent reliance on WEIRD normative cutoffs identified in this scoping review, these studies reveal a disconnect between the global use of motor assessments and the availability of culturally grounded norms. Our findings emphasize the need for culturally validated screening and assessment tools to ensure accurate identification of motor delays and prevent misclassification of normal cultural variations.

Cross‐cultural research indicates GM skill development is context‐dependent, highlighting the apparent role of the physical and sociocultural environments on the trajectory of GM skill development. Culturally embedded practices and contextual variables examined across included studies encompassed geographical context, caregiver and home environment characteristics, and caregiver practices. Our results are consistent with the integrated ecological model (An and Libertus 2025), as findings suggested positive association with GM development and parental beliefs in children's motor competence, caregiving behaviors that promote movement opportunities, greater home environment affordances and supportive sociocultural context. This review contributes to the limited body of evidence applying the novel integrated ecological theoretical framework and further supports the application of ecological theory and developmental niche framework to motor development.

Geographic location emerged as an important contextual factor associated with variation in GM development GM development, reflecting differences in social infrastructure, educational priorities and movement opportunities. Our results suggest population density alone does not necessarily facilitate or constrain motor development. Results of studies examining the differences in gross motor skills between rural and urban areas were variable. Facilitators of gross motor development in both areas may include an abundance of physical space to move in rural areas and greater access to resources such as organized sports in urban areas (Draper et al. 2017; Loureiro et al. 2024). Countries with higher health and education outcomes (e.g., HDI scores) may provide children with more consistent access to healthcare and early childhood education to support motor exploration (Fink et al. 2020). In contrast, lower national development indicators may constrain opportunities for movement through limited resources, reduced access to early education, or environmental barriers, which may adversely influence motor trajectories. These findings should be interpreted with caution though since only one study examined associations between gross motor development and HDI score and there is evidence of children living in countries with high HDI, such as the Netherlands, performing lower on gross motor measures than countries with lower HDI (Suir et al. 2019). Countries that integrate physical education into preschool curricula or prioritize movement‐based learning appeared to support higher levels of GM development (Richards et al. 2022; Brian et al. 2018; Van Waelvelde et al. 2008; D'Hondt et al. 2019; Giagazoglou et al. 2005). Overall, our results suggest that geographic context is associated with GM development through interconnected cultural, structural and policy‐driven systems rather than through location alone. It is important to note that contextual factors such as geographic location and SES do not by themselves constitute culture but serve as proxies for cultural context.

Caregiver and home characteristics were also identified as influential cultural and contextual factors that may interact with cultural beliefs and practices to shape a child's GM development. Higher SES level, higher parent education and greater home affordances were each positively associated with GM outcomes in early childhood. The interrelated nature of these characteristics implies that lower levels of one factor may consequently impact other factors. For example, lower parental education levels may result in lower income and less home affordances. Furthermore, parental education may be related to parental knowledge and beliefs surrounding development. However, associations between parent characteristics such as education level and income are not straightforward but rather nuanced as indicated by recent findings of high use of restrictive equipment for infants in families with high education levels and income in the United States (Alghamdi and Lobo 2025). This finding was multilayered, as high container use is associated with lower GM development but parental knowledge of development could possibly counteract the impacts of container use (Alghamdi and Lobo 2025). Caregiver relationships with young children appear to play a central role in all areas of development, including GM performance. Our results suggest intentional caregiver engagement, varied developmental positioning and movement opportunities to promote independent exploration are positively associated with GM development. Culture may shape caregiver choices regarding type and frequency of handling, positioning, movement exploration and subsequent exposure to different activities (e.g., dancing and biking) which may in turn relate to GM skill acquisition.

Based on our findings, we encourage clinicians and researchers to interpret motor milestones considering cultural caregiving practices and affordances rather than relying solely on WEIRD norms. To accomplish this, clinicians can use culturally responsive assessments of factors associated with motor development (e.g., Affordances in the Home Environment for Motor Development‐Infant Scale or AHEMD‐IS) (Caçola et al. 2011; Caçola et al. 2015). Informal assessment can include questions for caregivers on their knowledge and beliefs around development, caregiving practices and opportunities for movement. Clinicians and researchers can advocate for policies to support contextual factors during early development, including access to high‐quality, affordable early childhood education, universal health care, paid parental leave, urban design to optimize play and improved social infrastructure (World Health Organization UNCsF and World Bank Group 2018).

This scoping review has notable strengths. The search strategy was developed with a research librarian who provided rigorous, multidatabase searches to increase the likelihood of capturing the breadth of relevant literature. The search strategy also included grey literature sources, such as dissertations and theses to reduce publication bias and capture emerging research not yet published in peer‐reviewed journals. The updated search in 2025 ensured the review reflects the most current literature available. The JBI and PRISMA‐ScR checklist (Tricco et al. 2018; Peters et al. 2020) provided guidelines to support transparency and rigour throughout the process. OSF registration occurred before the study, reducing the risk of methodological bias. The large number of included studies permitted examination of a range of cultural variables influencing GM development across a diverse global landscape.

Several limitations should be considered when interpreting the findings. Article selection was restricted to English, which may have excluded research from other languages, contributing to possible underrepresentation of certain regions or cultures. This restriction is particularly important given the global scope of the review, as it may have increased representation of studies from WEIRD and English‐publishing contexts while underrepresenting culturally grounded research from non–English‐speaking regions. Future reviews should consider multilingual search strategies and inclusion of non–English publications to better capture worldwide cultural practices influencing GM development. Although the search strategy was developed with a research librarian, relevant studies using alternative terminology for cultural influences on development may not have been captured. Because our aim was to explore the breadth of existing literature, formal appraisal of methodological quality or risk of bias was not conducted. Consequently, the findings reflect the scope rather than the strength of the available evidence. Because the concept of culture was operationalized differently across studies (e.g., geography, SES, ethnicity and caregiving practices), direct comparisons were limited, complicating interpretation of cultural and contextual influences. In particular, variables such as SES and home affordances should be interpreted cautiously, as they may influence GM development across cultural settings and are not necessarily unique indicators of culture‐specific beliefs or practices. Substantial variation was also present across study methods and outcomes, preventing meta‐analysis of the findings.

This review identified several gaps in the literature that must be addressed to promote culturally responsive screening, assessment and intervention. The findings suggest a need to establish culturally grounded definitions of typical GM development through consensus work and cross‐cultural norming efforts and expand longitudinal designs to chart typical motor trajectories across diverse cultural contexts. Currently, the evidence base is largely cross‐sectional, offering snapshots of differences in motor development or cultural influences. Establishing culturally specific trajectories would better equip clinicians to distinguish motor delay from culturally expected patterns. Future research should employ multi‐level analytic approaches that connect individual, family, community and geographical variables within an integrated ecological and culturally grounded framework. This allows researchers to determine the influence of contextual factors within and across cultural settings. Additionally, the use of regression‐based approaches (e.g., logistic regression) may facilitate identification of cultural predictors of GM development rather than simple associations. Mixed‐methods designs are warranted to explore caregiver beliefs, routines and practices. Partnerships between local researchers and communities may strengthen the cultural relevance and interpretation of findings through approaches such as community‐based participatory research (Collins et al. 2018).

## 5 Conclusion

Culture is a central context associated with variation in typical GM development during early childhood. Our findings highlight the roles of geographical factors, SES, home affordances, parental perspectives and caregiving practices in relation to GM development in children from birth to 5 years. Clinicians and researchers are encouraged to assess young children's GM development through an integrated ecological lens and ensure use of culturally validated screening and assessment tools. Although the past three decades have generated extensive research on cultural and contextual influences on motor development, substantial gaps in the literature remain. Greater attention to culturally relevant developmental expectations, caregiving practices and contextual affordances, along with culturally validated measures and multi‐level longitudinal research, is essential for equitable developmental assessment and intervention worldwide.

## Author Contributions

**Jessica A. Silveira:** conceptualization, investigation, writing – original draft, writing – review and editing, visualization, methodology, project administration, resources. **Megan B. Flores:** conceptualization, investigation, writing – original draft, writing – review and editing, visualization, methodology, project administration, resources. **Jessica Barreca:** resources, project administration, writing – review and editing, visualization, methodology, conceptualization, investigation, writing – original draft. **Rebecca C. Hyde:** writing – review and editing, data curation, resources.

## Funding

The authors have nothing to report.

## Conflicts of Interest

The authors declare no conflicts of interest.

## Supporting information


**Appendix S1:** Preferred Reporting Items for Systematic Reviews and Meta‐Analyses Extension for Scoping Reviews (PRISMA‐ScR) checklist.

**Appendix S2:** Complete electronic search strategies for all databases searched in the scoping review.

**Appendix S3:** Data‐charting table for the 166 publications included in the scoping review.

## Data Availability

The data that support the findings of this study are available in the Supporting Information of this article.
