# Supplementary material for: Cultural Influences on Typical Gross Motor Development in Early Childhood: A Scoping Review
Source: Child Care Health Dev. 2026 Oct 15;52(6):e70364. doi: 10.1111/cch.70364 (PMC13671824; doi:10.1111/cch.70364)
Supplement: Supplementary file 1 [file CCH-52-e70364-s001.docx]

**Search Strategies for original searches (November 2023)**

**EBSCO’s CINAHL Ultimate, limited to 1994 to present.**

((MH "Child, Preschool") OR (MH "Infant") OR ("early childhood") OR (Pediatric) OR (toddler) OR (infant) OR (preschool*) OR (“young child”) OR (“young children”) OR (kindergarten*))

AND

((MH "Motor Skills") OR (MH "Sitting") OR (MH "Standing") OR (MH "Locomotion") OR (MH "Walking") OR (MH “Motor Activity”) OR (MH "Posture+") OR (“motor development”) OR (“Gross motor”) OR (“Motor milestones”) OR (“motor skills”) OR ("motor movement") OR ("motor performance"))

AND

((MH "Culture+") OR (MH "Minority Groups") OR (MH "Social Environment") OR (MH "Home Environment") OR (MH "Parental Behavior") OR (MH "Paternal Behavior") OR (MH "Maternal Behavior") OR (MH "Family Attitudes+") OR (MH "Intergenerational Relations") OR (MH "Parent-Child Relations+") OR (MH "Caregiver Attitudes") OR (MH "Child Care Providers") OR (MH "Race Factors") OR (MH "Geographic factors") OR (MH "Child Rearing") OR (cultur*) OR ("environmental factors") OR (“socioeconomic status”) OR (“parenting style”) OR (“ethnic values”) OR ((parent* or caregiver) AND expectation*) OR ((parent* or caregiver) AND practices) OR ((parent* or caregiver) AND handling))

AND

(LA "English")

**OVID’s APA PsycINFO (1987-present), limited to 1994-current**

(("120" or "140" or "160").ag. OR ("Child, Preschool" or "Infant").mh. OR ("preschool students" or "kindergarten students").sh. OR (Infant or "early childhood" or Pediatric or toddler or "young child" or "young children" or kindergarten* or preschool*).mp.)

AND

(("motor skills" or "sitting position" or "standing position" or running or locomotion or walking or “motor activity” or posture).mh. OR ("motor processes" or gait or jumping or locomotion or "motor control" or "motor performance" or running or walking or "motor skills" or "motor development" or "motor coordination" or posture or "gross motor skill learning").sh. OR ("gross motor" or "motor skills" or "motor development" or "motor performance" or "motor milestones" or "motor movement").mp.)

AND

((Culture or "Minority Groups" or "Social Environment" or "Home Environment" or "Paternal Behavior" or "Maternal Behavior" or "Intergenerational Relations" or "Parent-Child Relations" or "father-child relations" or "mother-child relations" or "Race Factors" or "Child Rearing").mh. OR (exp "racial and ethnic groups"/) OR ("Culture (Anthropological)" or "Sociocultural Factors" or "Cultural identity" or Traditions or "Cultural attitudes" or "Racial and ethnic differences" or "Cross cultural differences" or "Regional differences" or "Minority groups" or "Social environment" or "Home environment" or "Parental attitudes" or "Parenting style" or "childrearing practices" or "childrearing attitudes" or "parent child relations" or "mother child relations" or "father child relations" or "Parental expectations" or "Intergenerational Relations" or "Child care workers" or "Ethnic values").sh. OR (cultur* or "environmental factors" or "geographic factors" or "socioeconomic status" or "parental behavior" or (parent* adj3 expectation*) or (caregiver adj3 expectation*) or (parent* adj3 practices) or (caregiver adj3 practices) or (parent* adj3 handling) or (caregiver adj3 handling) or (family adj3 attitude*) or (parent* adj3 attitude*) or (caregiver adj3 attitude*) or (maternal adj3 attitude*) or (paternal adj3 attitude*)).mp.)

AND

(english.lg.)

**PubMed, limited to 1994 to present.**

("Child, Preschool"[Mesh] OR "Infant"[Mesh] OR "early childhood" OR Pediatric OR toddler OR Infant OR preschool* OR "young child" OR "young children" OR kindergarten*)

AND

("Motor Skills"[Mesh] OR "Posture"[Mesh] OR "Locomotion"[Mesh:NoExp] OR "Running"[Mesh] OR "Walking"[Mesh] OR "Motor Activity"[Mesh:NoExp] OR “motor development” OR “Gross motor” OR “Motor milestones” OR “motor skills” OR "motor movement" OR "motor performance")

AND

("Culture"[Mesh] OR "Minority Groups"[Mesh] OR "Social Environment"[Mesh:NoExp] OR "Home Environment"[Mesh] OR "Paternal Behavior"[Mesh] OR "Maternal Behavior"[Mesh] OR "Intergenerational Relations"[Mesh] OR "Parent-Child Relations"[Mesh] OR "Race Factors"[Mesh] OR "Child Rearing"[Mesh:NoExp] OR cultur* OR "environmental factors" OR “socioeconomic status” OR “parenting style” OR “ethnic values” OR “parental behavior” OR “geographic factors” OR ((parent*[Title/Abstract] OR caregiver[Title/Abstract]) AND expectation*[Title/Abstract]) OR ((parent*[Title/Abstract] OR caregiver[Title/Abstract]) AND practices[Title/Abstract]) OR ((parent*[Title/Abstract] OR caregiver[Title/Abstract]) AND handling[Title/Abstract]) OR ((family[Title/Abstract] or parent*[Title/Abstract] OR caregiver[Title/Abstract] OR maternal[Title/Abstract] OR paternal[Title/Abstract]) AND attitude*[Title/Abstract]))

AND

(English[Language])

**EBSCO’s ERIC, limited to 1994 to present.**

(DE("Preschool Children" OR "Young Children" OR "Toddlers" OR "Infants") OR "early childhood" OR Pediatric OR toddler OR Infant OR preschool* OR "young child" OR "young children" OR kindergarten*))

AND

(DE ("Psychomotor Skills" OR "Motor Development”) OR “motor skills” OR posture OR locomotion OR running OR walking OR sitting OR standing OR “motor activity” OR “motor development” OR “gross motor” OR “motor milestones” OR “motor performance” OR "motor movement")

AND

(DE("Culture" OR "Ethnic Groups" OR "Cultural Differences" OR "Ethnic Groups" OR "Minority Groups" OR "Social Environment" OR "Cultural Context" OR "Family Environment" OR "Parent Child Relationship" OR "Parent Influence" OR "Parenting Styles" OR "Racial Factors" OR "Child Rearing" OR "Cultural Differences" OR "Racial Differences" OR "Regional Characteristics" OR "Social Characteristics" OR "Parenting Styles" OR "Parent Attitudes" OR "Father Attitudes" OR "Mother Attitudes" OR "Caregiver Attitudes" OR "Family Attitudes" OR "Child Caregivers" OR "Socioeconomic Status") OR cultur* OR “home environment” OR "Intergenerational Relations" OR "environmental factors" OR “geographic factors” OR “socioeconomic status” OR “parenting style” OR “ethnic values” OR "paternal behavior" OR "maternal behavior" OR “parental behavior” OR “mother-child relations” OR “father-child relations” OR ((parent* or caregiver) AND expectation*) OR ((parent* or caregiver) AND practices) OR ((parent* or caregiver) AND handling))

AND

(LA "English")

**Web of Science Core Collection, limited to 1994-01-01 to 2023-12-30**

(TS=("young children" OR toddler OR infant OR "early childhood" OR Pediatric OR preschool* OR "young child" OR kindergarten*))

AND

(TS=("Motor Skills" OR Sitting OR Standing OR Locomotion OR Walking OR ”Motor Activity” OR running OR Posture OR “motor development” OR “Gross motor” OR “Motor milestones” OR "motor movement" OR "motor performance"))

AND

(TS=(Cultur* OR "Minority Groups" OR "Social Environment" OR "Home Environment" OR "Parental Behavior" OR "Paternal Behavior" OR "Maternal Behavior" OR "Intergenerational Relations" OR "Parent-Child Relations" OR “mother-child relations” OR “father-child relations” OR "Race Factors" OR "Child Rearing" OR childrearing OR "environmental factors" OR “socioeconomic status” OR “geographic factors” OR “parenting style” OR “ethnic values” OR ((parent* or caregiver) NEAR/3 expectation*) OR ((parent* or caregiver) NEAR/3 practices) OR ((parent* or caregiver) NEAR/3 handling) OR ((family or maternal or paternal or parent* or caregiver) NEAR/3 attitude*)))

AND

(LA=English)

**Scopus**

TITLE-ABS-KEY ({infant} OR {early childhood} OR pediatric OR toddler OR {young child} OR {young children} OR kindergarten* OR preschool*)

AND

TITLE-ABS-KEY({Motor Skills} OR posture OR locomotion OR running OR walking OR sitting OR standing OR {motor activity} OR {motor development} OR {gross motor} OR {motor milestones} OR {motor movement} OR {motor performance})

AND

TITLE-ABS-KEY(Cultur* OR {Minority Groups} OR {Social Environment} OR {Home Environment} OR {Parental Behavior} OR {Paternal Behavior} OR {Maternal Behavior} OR {Intergenerational Relations} OR “Parent-Child Relations” OR “father-child relations” OR “mother-child relations” OR {Race Factors} OR {Child Rearing} OR childrearing OR “parenting style” OR “ethnic values” OR {environmental factors} OR {geographic factors} OR {socioeconomic status} OR ((parent* or caregiver) W/3 expectation*) OR ((parent* or caregiver) W/3 practices) OR ((parent* or caregiver) W/3 handling) OR ((parent* or caregiver) W/3 attitude*) OR ((family or maternal or paternal or parent* or caregiver) W/3 attitude*))

AND

PUBYEAR > 1993

AND

LANGUAGE(English)

**ProQuest Dissertations & Theses Global, Limited to English**

noft("young children" OR toddler OR infant OR "early childhood" OR Pediatric OR preschool* OR "young child" OR kindergarten*)

AND

noft("Motor Skills" OR Sitting OR Standing OR Locomotion OR Walking OR ”Motor Activity” OR running OR Posture OR “motor development” OR “Gross motor” OR “Motor milestones” OR "motor movement" OR "motor performance")

AND

noft(Cultur* OR "Minority Groups" OR "Social Environment" OR "Home Environment" OR "Parental Behavior" OR "Paternal Behavior" OR "Maternal Behavior" OR "Intergenerational Relations" OR "Parent-Child Relations" OR “father-child relations” OR “mother-child relations” OR "Race Factors" OR "Child Rearing" OR childrearing OR "environmental factors" OR “socioeconomic status” OR “parenting style” OR “ethnic values” OR “geographic factors” OR ((parent* or caregiver) NEAR/3 expectation*) OR ((parent* or caregiver) NEAR/3 practices) OR ((parent* or caregiver) NEAR/3 handling) OR ((family or maternal or paternal or parent* or caregiver) NEAR/3 attitude*))

AND

YR(>1993)

**Search Strategies for updated searches (February? 2025)**

Since access to OVID APA PsycInfo is no longer available, the search was translated for use in EBSCO APA PsycInfo. All other databases will use the same search except the dates searched will only include November 2023 to present.

**APA PsycInfo EBSCO, limited to November 2023-present**

(MA ("Child, Preschool" or "Infant") OR DE ("preschool students" or "kindergarten students") OR (Infant or "early childhood" or Pediatric or toddler or "young child" or "young children" or kindergarten* or preschool*))

AND

(MA ("motor skills" or "sitting position" or "standing position" or running or locomotion or walking or “motor activity” or posture) OR DE ("motor processes" or gait or jumping or locomotion or "motor control" or "motor performance" or running or walking or "motor skills" or "motor development" or "motor coordination" or posture or "gross motor skill learning") OR ("gross motor" or "motor skills" or "motor development" or "motor performance" or "motor milestones" or "motor movement"))

AND

(MA (Culture or "Minority Groups" or "Social Environment" or "Home Environment" or "Paternal Behavior" or "Maternal Behavior" or "Intergenerational Relations" or "Parent-Child Relations" or "father-child relations" or "mother-child relations" or "Race Factors" or "Child Rearing") OR DE ("Racial and Ethnic Groups" OR “African Cultural Groups" OR "Asians" OR "Black People" OR "European Cultural Groups" OR "Indigenous Populations" OR "Latinos/Latinas" OR "Middle Eastern and North African Cultural Groups" OR "Multiracial" OR "North American Cultural Groups" OR "Oceanian Cultural Groups" OR "People of Color" OR "Romanies" OR "South American Cultural Groups" OR "White People") OR DE ("Culture (Anthropological)" or "Sociocultural Factors" or "Cultural identity" or Traditions or "Cultural attitudes" or "Racial and ethnic differences" or "Cross cultural differences" or "Regional differences" or "Minority groups" or "Social environment" or "Home environment" or "Parental attitudes" or "Parenting style" or "childrearing practices" or "childrearing attitudes" or "parent child relations" or "mother child relations" or "father child relations" or "Parental expectations" or "Intergenerational Relations" or "Child care workers" or "Ethnic values") OR (cultur* or "environmental factors" or "geographic factors" or "socioeconomic status" or "parental behavior" or (parent* W3 expectation*) or (caregiver W3 expectation*) or (parent* W3 practices) or (caregiver W3 practices) or (parent* W3 handling) or (caregiver W3 handling) or (family W3 attitude*) or (parent* W3 attitude*) or (caregiver W3 attitude*) or (maternal W3 attitude*) or (paternal W3 attitude*)))

AND

(LA “English”)
